# Markers of Mitochondrial Function and DNA Repair Associated with Physical Function in Centenarians

**DOI:** 10.3390/biom14080909

**Published:** 2024-07-26

**Authors:** Ines Sanchez-Roman, Beatriz Ferrando, Camilla Myrup Holst, Jonas Mengel-From, Signe Hoei Rasmussen, Mikael Thinggaard, Vilhelm A. Bohr, Kaare Christensen, Tinna Stevnsner

**Affiliations:** 1 Department of Molecular Biology and Genetics, Aarhus University, 8000 Aarhus, Denmark; camilla.myrup.holst@mbg.au.dk (C.M.H.); 2 Danish Aging Research Center, Department of Public Health, University of Southern Denmark, 5230 Odense, Denmark; 3 Facultad de Humanidades y Ciencias Sociales, Universidad Isabel I, 09003 Burgos, Spain; beatriz.ferrando@ui1.es (B.F.); 4 Epidemiology, Biostatistics and Biodemography, University of Southern Denmark, 5230 Odense, Denmark; jmengel-from@health.sdu.dk (J.M.-F.); signe.hoei.rasmussen@rsyd.dk (S.H.R.); mthinggaard@health.sdu.dk (M.T.); kchristensen@health.sdu.dk (K.C.); 5 Geriatric Research Unit, Department of Clinical Research, University of Southern Denmark, 5230 Odense, Denmark; 6 Department of Cellular and Molecular Medicine, University of Copenhagen, 2200 Copenhagen, Denmark; vbohr@sund.ku.dk

**Keywords:** centenarians, mitochondria, DNA repair, grip strength, physical function

## Abstract

Mitochondrial dysfunction and genomic instability are key hallmarks of aging. The aim of this study was to evaluate whether maintenance of physical capacities at very old age is associated with key hallmarks of aging. To investigate this, we measured mitochondrial bioenergetics, mitochondrial DNA (mtDNA) copy number and DNA repair capacity in peripheral blood mononuclear cells from centenarians. In addition, circulating levels of NAD+/NADH, brain-derived neurotrophic factor (BDNF) and carbonylated proteins were measured in plasma and these parameters were correlated to physical capacities. Centenarians without physical disabilities had lower mitochondrial respiration values including ATP production, reserve capacity, maximal respiration and non-mitochondrial oxygen-consumption rate and had higher mtDNA copy number than centenarians with moderate and severe disabilities (*p* < 0.05). In centenarian females, grip strength had a positive association with mtDNA copy number (*p* < 0.05), and a borderline positive trend for activity of the central DNA repair enzyme, APE 1 (*p* = 0.075), while a negative trend was found with circulating protein carbonylation (*p* = 0.07) in the entire cohort. Lastly, a trend was observed for a negative association between BDNF and activity of daily living disability score (*p* = 0.06). Our results suggest that mechanisms involved in maintaining mitochondrial function and genomic stability may be associated with maintenance of physical function in centenarians.

## 1. Introduction

Aging is associated with loss of physical function, which is critical for the maintenance of independence and autonomy at old age and essential to preserve quality of life [1]. Many measures have been optimized to study physical abilities in very old age [2,3]. However, the molecular mechanisms that underlie age-related decline in physical performance and associated health consequences are not fully understood. Centenarians represent an extraordinary subpopulation of successful aging individuals, but they show a wide heterogeneity regarding both physical and cognitive decline. These two age-related outcomes may be influenced by several common factors, including sociodemographic and genetic differences [4,5,6]. We have previously reported that mitochondrial respiration is well preserved in centenarians and, like APE1 endonuclease activity, brain-derived neurotrophic factor (BDNF) and NAD+/NADH levels, it is associated with the maintenance of cognitive abilities at very old age [7,8]. This supports the hypothesis that mitochondrial dysfunction and genomic instability are molecular hallmarks of aging also at the highest ages (reviewed in [9,10,11]).

The influence of mitochondrial function on aging in skeletal muscle has been investigated to some extent. Most studies suggest that aging is associated with a decline in mitochondrial respiratory capacity and increased reactive oxygen species (ROS) production (reviewed in [12,13]). In line with previous findings, mitochondrial dysfunction has been linked to the development of age-related pathologies, including sarcopenia and frailty [14]. Hence, mitochondrial function seems to play an important role in maintaining physical function with age. Since multiple studies have indicated that peripheral blood mononuclear cells (PBMCs) mirror the bioenergetic characteristics of many tissues, investigations into mitochondria in PBMCs have emerged as a viable method to evaluate mitochondrial health [15,16]. Previous studies support the positive association between parameters of mitochondrial bioenergetic capacity in PBMCs with gait speed as well as with muscle function and strength in obese adults [17]. This highlights how mitochondrial respiration measured in PBMCs may be indicative of both aerobic and anaerobic activities in human studies. Mitochondrial DNA (mtDNA) content has also served as a biomarker for mitochondrial function, with the majority of studies identifying a negative association between mtDNA copy number and age [18]. Interestingly, Mengel-From and coworkers showed an association between low mtDNA copy number in blood and higher mortality as well as poor health (both physical and cognitive decline) at old age [19]. Likewise, another study observed an association between decreased levels of mtDNA copy number and increased frailty across two cohorts of elderly adults [20]. Overall, these studies suggest that high mtDNA copy number is associated with better health and survival in elderly individuals.

Accumulation of oxidative DNA damage occurs during aging and contributes to genomic instability, a widely recognized hallmark of aging [9]. Base Excision Repair (BER) stands out as a major DNA repair pathway tasked with eliminating oxidative DNA lesions, thus assuming a pivotal role in safeguarding genome stability. Briefly, BER is initiated by a DNA glycosylase that generates an apurinic/apyrimidinic (AP or abasic) site, which is processed by an AP endonuclease. In humans, the major AP endonuclease is AP endonuclease 1 (APE1), which has also been identified as both the rate-limiting and critical step of the process [21]. APE1 creates a single-strand break, and the resulting gap is processed by the downstream BER pathway enzymes. Altered BER capacity and accumulation of oxidative DNA damage have been observed during aging and in age-associated neurodegenerative diseases [22] as well as in old adults suffering from frailty [23]. Apart from its crucial function in the BER pathway, APE1 is also reported to interact with and stimulate the activity of several other key BER proteins and to be involved in activating some transcription factors [24]. Specifically, APE1 has been associated with aging and neurodegenerative diseases, such as Alzheimer’s disease [25]. Interestingly, the expression of APE1 in rat brain has been shown to be enhanced by an exercise-induced neurotrophic factor (BDNF) [26]. Importantly, BDNF declines with age [27] and is associated with memory consolidation and cognitive function. Besides its known protective role against neurodegenerative diseases [28], BDNF has been linked to muscle strength [29] with decreased levels associated with frailty in old adults [30].

Another factor pertinent to mitochondrial function and associated with the aging process is the indispensable coenzyme nicotinamide adenine dinucleotide (NAD+). NAD+, together with NADH, are central to energy metabolism. NAD+/NADH stimulates mitochondrial functions and DNA repair pathways, which are critical for maintaining healthy aging. Interestingly, lower NAD+ levels have been observed in age-related diseases, including some which involve cognitive and physical decline [31]. Restoring NAD+ levels during aging has emerged as an interesting therapeutical approach, improving cellular energetics and functional capacity in rodents [32].

While the age-related factors mentioned above have undergone extensive examination within the fields of aging research, it remains unclear whether they can serve as indicators of preservation of physical function at very old age. In this study, we evaluate the physical capacity in a Danish cohort comprising centenarians born in 1915 and examine potential correlations with key biomarkers of aging in their blood. These biomarkers include mitochondrial bioenergetics, mtDNA copy number, APE1 quantity and activity, BDNF levels, NAD+/NADH levels and parameters related to oxidative damage.

## 2. Materials and Methods

### 2.1. Study Population

This study included centenarians born between 1 January 1915 and 31 December 1915, alive on their 100th birthday and living in the western part of Denmark, with no exclusion criteria (Table 1). Informed consent was obtained from all participants and the study was conducted according to the guidelines laid down in the Declaration of Helsinki. All procedures involving human subjects were approved by the Committee on Health Research Ethics (trial number S-20140099) and the Danish Data Protection Agency (registration number 2016-41-4552). Of the 303 eligible persons, 238 participated (79%). Only participants who were able to understand the concept of the study could participate directly (n = 185) (including tests), while others were interviewed by proxy (n = 53), but from the latter no blood samples were drawn and neither physical nor cognitive testing was performed. In total, 135 participants had a blood sample drawn. The assessment procedure has been described in detail previously [7,33].

### 2.2. Physical Function Assessment

Physical functioning was assessed through different approaches, including both a physical performance test (grip strength) and an activities of daily living score (ADL disability score). Grip strength was evaluated according to [33,34]. Briefly, grip strength in kilograms was measured using a Smedley dynamometer (TTM; Tokyo, Japan). To measure maximal strength, the handle width was adjusted to fit the hand size, ensuring the second phalanx rested against the inner stirrup. Grip strength is influenced by elbow position, with greater strength observed when the elbow is fully extended. For our measurements, we required the elbow to be positioned at 90 degrees and the upper arm to be kept tight against the trunk. A series of three measurements was taken, with brief pauses between each, and the highest value was used as the estimate of maximal grip strength. In all, grip strength measures were obtained from 126 participants (Table 1). Briefly, five Basic Activities of Daily Living (BADL) were derived from self-reports of eleven physical tasks. Disability was measured as a disability score based on the five BADL tasks presented in hierarchical order of difficulty (most difficult task first): bathing, dressing, toileting, transferring and feeding. To calculate a disability score, each of the five BADL tasks was changed to binary variables: “1” can do the activity independently and “0” can do the activity with help or cannot do the activity at all. Thereby a total score between 0 and 5 could be obtained, which was divided into three groups: 0–2 (severely disabled), 3–4 (moderately disabled) and 5 (nondisabled).

### 2.3. Cognitive Ability Assessment

A Mini-Mental State Examination (MMSE) test was employed to assess cognition as previously described [7,33]. The MMSE test is a test divided into two sections: the first section covers memory, orientation and attention (with a maximum score of 21), while the second section assesses the ability to follow verbal and written commands, name objects, spontaneously write a sentence and copy a complex polygon (with a maximum score of 9). The maximum possible MMSE score is 30, which indicates intact cognitive function. The cognitive characteristics of the selected cohort of Danish centenarians born in 1915 have been outlined in [7]. Ninety-five percent of the centenarians completed the MMSE, with scores ranging from 5 to 29, indicating considerable diversity in cognitive abilities within the centenarian population.

### 2.4. Blood Sampling, PBMCs and Plasma Isolation

Eight milliliters of venous blood were collected from all participants who consented to donate using BD Vacutainer Cell Preparation Tubes (CPT) with sodium citrate (BD Biosciences, Herlev, Denmark). Twenty-four hours after blood collection, PBMCs were isolated as outlined by [7,35], and plasma was separated as the upper layer after the initial centrifugation. Cells were resuspended in XF assay medium (Nordic, Glostrup, Denmark) supplemented with 25 mM glucose (Merck, Darmstadt, Germany) and 4 mM pyruvate (Merck, Darmstadt, Germany), pH 7.4, and diluted to 2 million cells per mL. 

### 2.5. Analysis of Mitochondrial Function in PBMCs

Mitochondrial function in PBMCs was assessed using a Seahorse XF extracellular flux analyzer, which provides real time measurements of oxygen consumption (OCR) and extracellular acidification (ECAR) [36]. In brief, OCR serves as an indicator of mitochondrial respiration while ECAR primarily measures lactic acid production during glycolytic energy metabolism. Blood cell bioenergetics has been extensively studied as an indicator of systemic mitochondrial dysfunction [15,37]. Consequently, bioenergetics analysis has been performed as described in [7]. Specifically, PBMCs (300,000 cells/well) were attached to 24 well respiration plates precoated with 100 µg/mL Cell-Tak (VWR, Soeborg, Denmark) by centrifugation and experiments were performed with a final volume of 660 µL/well of XF assay medium (Nordic, Glostrup, Denmark). Following an extensive optimization procedure detailed in [7], OCR measurements were obtained using two separate assays on the same individual PBMCs: one set of cells received an initial injection of 1 μM oligomycin (Merck, Darmstadt, Germany) followed by the inhibitors 2 μM rotenone (Merck, Darmstadt, Germany) and 2 μM Antimycin A (Merck, Darmstadt, Germany) while the other set of cells received 0.6 μM FCCP (Merck, Darmstadt, Germany) first, followed by 2 μM rotenone and 2 μM Antimycin A. This approach minimized variation and underestimation of maximal respiration and reserve capacity, which has been observed with the classical method [38]. Data were normalized to protein concentration by aspirating the medium after the assay and lysing the cells in 30 µL/well of RIPA buffer (150 mM NaCl, 1% Triton, 0.1% SDS, 10 mM Tris pH 8 and 0.5% Sodium deoxycholate; all from Merck, Darmstadt, Germany), then storing at −20 °C. Protein concentration was determined using a BCA assay with a bovine serum albumin (BSA) (Merck, Darmstadt, Germany) standard curve as reference.

### 2.6. mtDNA Copy Number from PBMCs

DNA was isolated from PBMCs using the standard Qiagen Puregene protocols. The mtDNA copy number was measured as previously described [19] in triplicates. In summary, the assay employs real time polymerase chain reaction (PCR) and SYBR^®^ Green technology (ThermoFisher Scientific, Roskilde, Denmark). The mtDNA copy number was calculated using the formula 2(CtBG median − CtND median) as detailed elsewhere [19].

### 2.7. Whole Cell Extract (WCE) from PBMCs

Whole cell extract (WCE) preparation from PBMCs was carried out following established procedures [39]. PBMCs were thawed at room temperature (RT), washed in RPMI with 10% FBS (ThermoFisher Scientific, Roskilde, Denmark) and resuspended to a concentration of approximately 20,000 cells/µL in a buffer containing 1 mM EDTA, 50 mM Tris-HCl (pH 7.0), 0.5 mM DTT, 0.1 mM spermine, 0.5 mM spermidine and a protease inhibitor cocktail (all from Merck, Darmstadt, Germany). The PBMCs were incubated for 30 min on ice, followed by freeze–thaw lysis (35 min incubation in dry-ice-ethanol slurry followed by 5 min at 30 °C). Proteins were extracted by adding KCl (Merck, Darmstadt, Germany) to a final concentration of 220 mM and incubating for 30 min on ice. Cell debris was removed by centrifugation at 11,000× g for 15 min at 4 °C. Glycerol (Merck, Darmstadt, Germany) was added to a final concentration of 10%. Protein concentration was determined using a Bradford assay with bovine serum albumin (BSA) (Merck, Darmstadt, Germany) as a standard, following the manufacturer’s protocol. WCE was stored at −80 °C until further analysis. Both WCE and PBMCs can be stored at −80 °C for an extended period without detectable modifications in the BER capacity [40].

### 2.8. Radiolabelling of Oligonucleotides

All oligonucleotides were obtained from Merck (Darmstadt, Germany). Oligonucleotides containing the stable AP site analogue tetrahydrofuran derivate (THF) with the sequence 5′-ATA TAC CGC GG(THF) CGG CCG ATC AAG CTT ATT-3′ and the corresponding control oligonucleotide damage-free with sequence 5′-ATA TAC CGC GGC CGG CCG ATC AAG CTT ATT-3′ were 5′ labelled with 32P and annealed to their complementary oligonucleotide (5′-AAT AAG CTT GAT CGG CCG GCC GCG GTA TAT-3′). Single-stranded oligonucleotides used as a DNA size marker were similarly labelled. Moreover, 100 ng oligonucleotide was incubated with 100 µCi 32P-γ-ATP (Nordic, Ballerup, Denmark) and 20 units of T4 polynucleotide kinase (ThermoFisher Scientific, Roskilde, Denmark) for 90 min at 37 °C in 20 µL of 1× forward buffer A (ThermoFisher Scientific, Roskilde, Denmark) followed by 1 min incubation at 90 °C. Unincorporated 32P-γ-ATP was removed by size exclusion on a G-50 column (Merck, Darmstadt, Germany). The labelled oligonucleotides were annealed to a 500 ng non-labelled complementary strand in a buffer that contains 175 mM KCl (Merck, Darmstadt, Germany) and 90 mM EDTA (Merck, Darmstadt, Germany) by heating to 90 °C for 5 min, followed by gradual cooling to room temperature overnight. Complete annealing was confirmed by gel electrophoresis on a 20% native polyacrylamide gel, 20% ProtoGel (BioNordika, Herlev, Denmark), 0.075% APS (Merck, Darmstadt, Germany), 1× TBE buffer (ThermoFisher Scientific, Roskilde, Denmark) and 5 µM TEMED (Merck, Darmstadt, Germany) and visualized by phoshoimaging (Bio-Rad, Copenhagen, Denmark). The amount of labelled oligonucleotide remaining after the column was quantified using Image Quant (Bio-Rad, Copenhagen, Denmark). 

### 2.9. APE1 Endonuclease Activity Assay of WCE from PBMCs

APE1 endonuclease activity assay was conducted following established procedures [41]. To determine the optimal concentration of WCE for incision activity within the linear range, 1–400 ng of WCE was incubated with 30 fmol of 5′-32P-labelled double-stranded oligonucleotide with or without the THF modification for 15 min at 37 °C in a 20 µL reaction containing 0.4 mM EDTA, 75 mM Tris-HCl (pH 7.0), 9 mM MgCl2, 89 mM KCl, 5% glycerol and 2.5 µg/µL BSA (All from Merck, Darmstadt, Germany). The reaction was stopped by adding 20 µL of 2× formamide loading buffer (80% formamide, 1 mg/mL bromophenol, 10 mM EDTA, 1 mg/mL xylene cyanol FF), followed by 10 min incubation at 90 °C (All from Merck, Darmstadt, Germany). Substrate and product were separated on a 20% denaturing polyacrylamide gel: 1× TBE buffer, 0.75M UreaGel Diluent (BioNordika, Herlev, Denmark), UreaGel Concentrate (20% acrylamide, 0.75M urea) (BioNordika, Herlev, Denmark), 0.075% APS (Merck, Darmstadt, Germany) and 5 µM TEMED (Merck, Darmstadt, Germany) and visualized by phoshoimaging. APE1 endonuclease activity was quantified using ImageQuant (Bio-Rad, Copenhagen, Denmark) and the percentage activity was calculated as the amount of radioactivity present in the product band relative to the total radioactivity in both the product and uncleaved substrate band. Based on the WCE titration, 20 ng and 50 ng of WCE were chosen for subsequent assays to measure APE1 endonuclease activity. The activity was expressed as the average amol cleaved product per ng WCE per minute. An internal control sample was included in each assay, and all measurements were normalized to the internal control to minimize inter-assay variation.

### 2.10. Protein Carbonylation of Circulating Plasma Proteins

Oxidative modifications of plasma proteins were analyzed by immunoblot detection of protein carbonyl groups using the ‘OxyBlot’ protein oxidation detection kit following the manufacturer’s instructions (Merck, Darmstadt, Germany). Moreover, 10 μg of plasma proteins were derivatized by 2,4-dinitrophenylhydrazine and separated on Tris-Glycine SDS-PAGE (5% stacking, 12% separating) for 3 h at 120 V. After transfer, PVDF membranes were blocked with 1% BSA (in phosphate buffered saline containing 0.05% Tween-20) and incubated with anti-dinitrophenylhydrazone antibody (Merck, Darmstadt, Germany) (1:300 working dilution) overnight at 4 °C. Subsequently, membranes were incubated with a secondary antibody: goat anti-rabbit IgG (horseradish peroxidase-conjugated; Merck, Darmstadt, Germany) diluted 1:300 in blocking solution for 1 h at room temperature. Protein detection was conducted with ECL prime western blotting detection reagent Merck, Darmstadt, Germany) and Amersham Imager 600 (GE Healthcare, Amersham). Quantification of total protein and protein carbonyls was performed by densitometry of the immunoblots and of the membranes stained with Red Ponceau S solution (Merck, Darmstadt, Germany), respectively. The ratio between total protein carbonyls and total protein was determined, and the data were normalized to the average intensity of the internal control duplicates on each membrane to minimize inter blot variability.

### 2.11. ELISA Analysis of Circulating Plasma Samples

BNDF and NAD+/NADH levels in plasma were measured using the colorimetric BDNF (Ab99978) and NAD+/NADH (Ab65348) kit assays, respectively, following the manufacturer’s protocols (Merck, Darmstadt, Germany). Duplicate sample absorbances were measured at 450 nm on a microplate reader. Absorbance values of individual samples were directly compared within each plate and normalized against a standard positive control to determine concentration. Data normalization was performed based on the average intensity of duplicate internal control samples within each microplate to minimize variability across plates.

### 2.12. Statistical Analyses

Statistical evaluation was performed using STATA (Version 13) and Graph Pad PRISM (version 6.01, La Jolla, CA, USA). Data are presented as mean ± SD (Standard Deviation) or median ± IQR (Interquartile range, as indicated). Normal distribution of variables was evaluated by Shapiro–Wilk test and non-parametric tests were used when appropriate. Equal variance of the groups was tested by an F test. Linear regression analysis or Pearson correlation was conducted to test the association between the different variables. A median quantile regression or Spearman rank correlation was performed when there were no normal distributed data. Three-group comparison was carried out with one-way ANOVA (assuming unequal or equal variance in normal distributed variables) or Kruskal–Wallis test (in non-normal distributed variables). Tukey (after ANOVA) and Dunn’s (after Kruskal–Wallis test) post hoc methods were used for multiple comparison with Bonferroni correction. *p*-values < 0.05 were considered statistically significant.

## 3. Results

### 3.1. Association between Cognitive Measurements and Physical Performance in Centenarians

**Figure 1:**
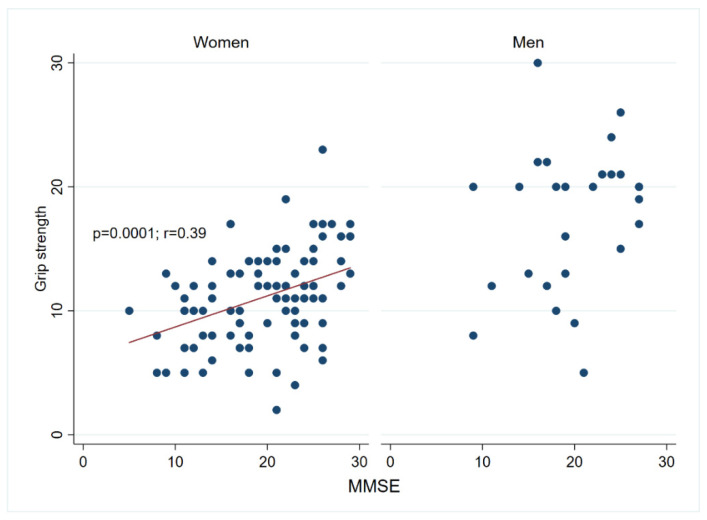
Grip strength correlates positively with cognition, measured as MMSE (Mini Mental State Examination) in female centenarians. The association between cognitive scores, measured as MMSE and grip strength was tested using a Pearson correlation in women (n = 93; *p* = 0.0001, r = 0.39) and men (n = 27; *p* = 0.18, r = 0.264). A tendency line is plotted when the correlation analysis is significant.

**Figure 2:**
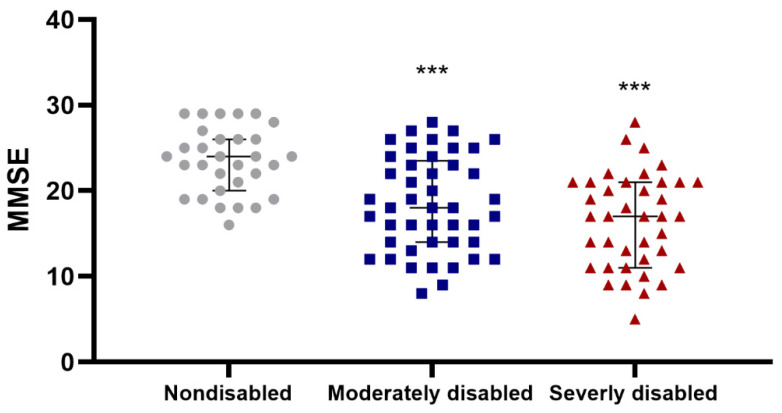
Higher cognitive score (MMSE) is associated with a lower ADL disability score. The association between the cognitive measurement, MMSE and the ADL disability score was assessed by a Kruskal–Wallis test (n = 120; *p* = 0.0001) followed by a Dunn test pairwise comparison with Bonferroni correction (*** indicates differences in comparison to nondisabled; *** *p*< 0.001). Median ± IQR (interquartile range) is shown.

Details regarding the study population are provided in Table 1. Most of the centenarians were females (78%). A comprehensive description of the cohort has been previously reported elsewhere [33]. Grip strength exhibited a sex-based association, with males demonstrating higher levels than females, as expected. However, such a discrepancy based on sex was not evident in the ADL disability score (Supplementary Table S1). Both assessments are well validated and have previously been employed to evaluate physical performance in populations of aged individuals [42,43,44] and in our cohort, they also exhibit a significant association even after adjusting for sex (Supplementary Table S1). Cognitive measurements were also evaluated in this cohort and have previously been reported in [7]. Prior research conducted among very old individuals has found a notable correlation between cognition and physical performance [2,6,45]. To verify whether this association holds true within our cohort, we conducted an analysis of physical parameters and cognitive measurements. This revealed a positive association between the cognitive values measured as MMSE (Mini Mental State Examination) score and grip strength and a negative association between MMSE and ADL disability score. Thus, we noted a robust and statistically significant positive correlation (*p* < 0.001) between grip strength and MMSE scores among females (n = 93), whereas such a correlation did not reach significance among the smaller sample of males (n = 27) (Figure 1). In addition, the MMSE score was higher in nondisabled centenarians according to the ADL disability score, while moderately and severely disabled centenarians showed lower MMSE values (Figure 2; *p* < 0.001; n = 120).

### 3.2. Mitochondrial Function in PBMCs and Physical Performance in Centenarians

**Figure 3:**
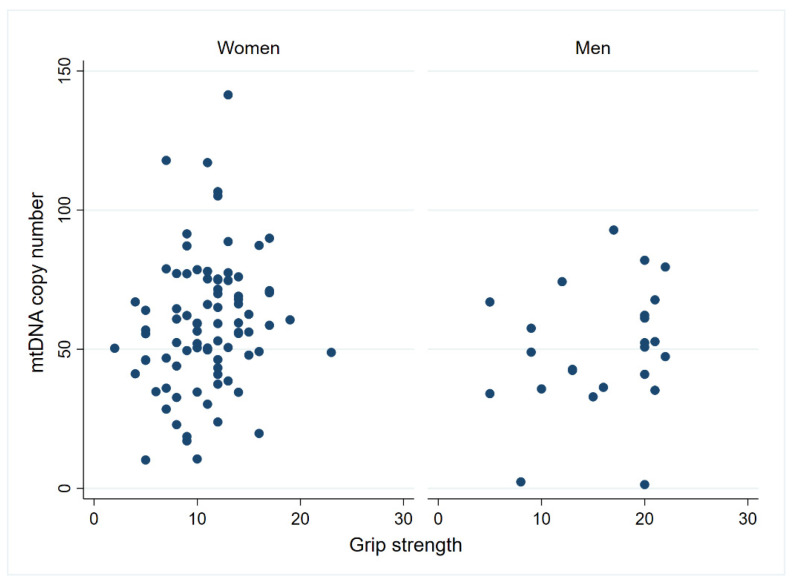
Mitochondrial DNA copy number correlates positively with grip strength in female centenarians. The association between grip strength and mtDNA copy number was tested using a Spearman rank correlation in women (n = 84; *p* = 0.04; rho = 0.22) and men (n = 24; *p* = 0.25; rho = 0.24).

**Figure 4:**
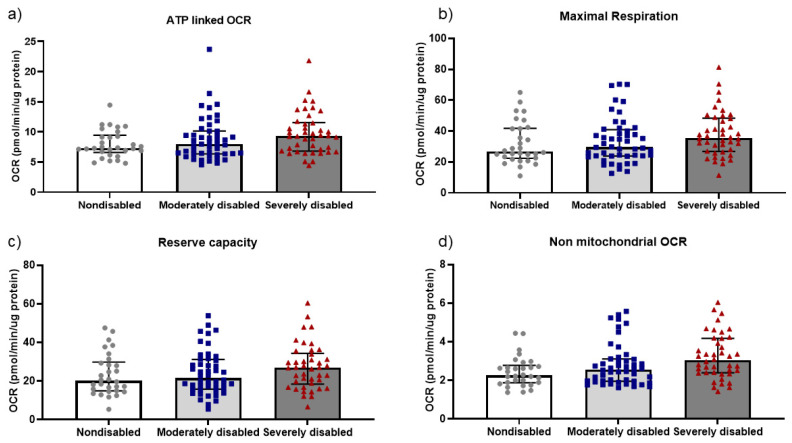
(**a**) ATP linked OCR, (**b**) Maximal Respiration, (**c**) Reserve capacity and (**d**) Non-mitochondrial OCR are associated positively with ADL disability score. The association between mitochondrial parameters and ADL disability score was assessed by a quantile regression of the median (Supplementary Table S3) in women (n = 84) and men (n = 25) after adjustment by sex (**a**) *p* = 0.03; coefficient = 1.0; (**b**) *p* = 0.03; coefficient = 4.58; (**c**) *p* = 0.01; coefficient = 4.18; (**d**) *p* = 0.01; coefficient = 0.19. For a description of the statistical analyses performed, see Supplementary Table S3. Each value represents the mean of 2–5 replicates. Median ± IQR (interquartile range) is shown. Bars indicate median.

**Figure 5:**
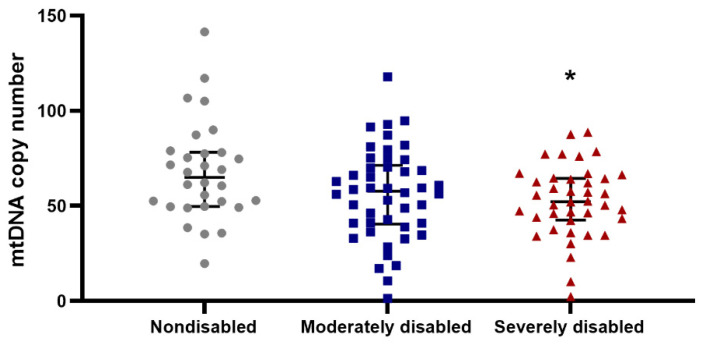
Mitochondrial DNA copy number is negatively associated with the ADL Disability score. Centenarians nondisabled according to the ADL disability score showed higher values of mtDNA copy number in their PBMCs in comparison to centenarians moderately and severely disabled. The association between the disability score and mtDNA copy number in women (n = 84) and men (n = 24) was assessed by a Kruskal–Wallis test (*p* = 0.04) followed by Dunn’s multiple comparison test with Bonferroni correction (* indicates differences to nondisabled) and a quantile regression of the median (Supplementary Table S3).

Mitochondrial respiration and mtDNA copy number were investigated in the centenarian cohort as indicators of mitochondrial functionality. Upon examining mitochondrial respiration, no significant associations between grip strength and mitochondrial parameters in PBMC were observed (Supplementary Table S2). Conversely, mitochondrial DNA copy number exhibited a significant positive correlation with grip strength when correlations were explored within the female cohort (n = 84; *p* < 0.05). A similar correlation was, however, not significant for the male cohort (n = 24), which may be due to the low number of male observations (Figure 3). Upon scrutinizing the disability score, we identified a positive association between the following mitochondrial parameters: ATP-linked OCR, Maximal Respiration, Reserve Capacity, non-mitochondrial OCR and Basal ECAR when compared to the ADL disability score, even after adjusting for sex (Supplementary Table S3 and Figure 4). Additionally, a negative association was observed between mitochondrial DNA copy number and disability score when evaluating the entire cohort (Supplementary Table S3 and Figure 5) (n = 108; *p* < 0.05). Thus, nondisabled centenarians showed higher mitochondrial DNA copy number and lower values of the cited mitochondrial parameters than moderately and severely disabled ones.

### 3.3. APE1 Endonuclease Activity in Centenarian PBMCs Is Associated with Physical Performance

**Figure 6:**
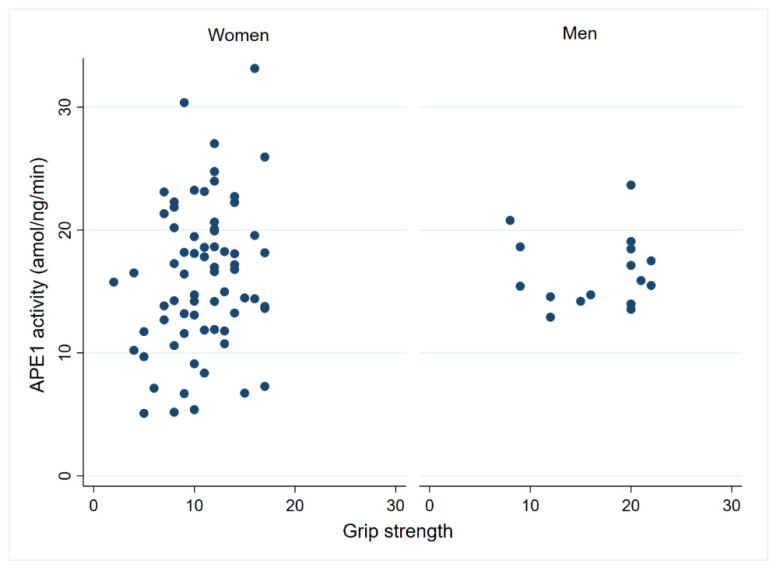
PBMC APE1 activity correlation with grip strength in female centenarians. The association between grip strength and APE1 activity was tested using a Pearson correlation (*p* = 0.07; r = 0.21 in females; n = 68 and *p* = 0.92, r = 0.02 in males; n = 16).

APE1 endonuclease activity has previously been linked to the preservation of cognitive capacity in centenarians [7]. To investigate whether it also relates to the maintenance of physical function, we assessed its association with the physical performance test conducted in the centenarians. Among female centenarians, there was a suggestive correlation (n = 68; *p* = 0.07) between APE1 activity and grip strength (Figure 6). However, this association was not observed in the male cohort. Additionally, no significant differences were found when comparing the ADL disability score with APE 1 activity or protein levels (Supplementary Table S4).

### 3.4. Circulating NAD+/NADH Levels’ Association with Physical Performance

Next, NAD+/NADH levels were assessed in the plasma of the centenarians. No significant correlations were found between grip strength and circulating NAD+/NADH levels within the entire cohort, nor between ADL disability score and circulating NAD+/NADH levels (Supplementary Table S4). In addition, we did not observe any correlations when females or males were analyzed separately.

### 3.5. Physical Abilities and Oxidative Stress Markers in Centenarians

**Figure 7:**
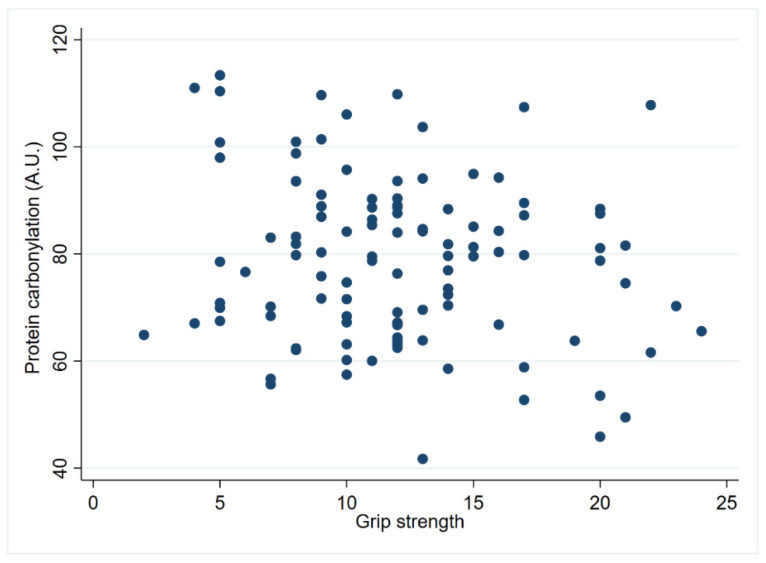
Protein carbonylation correlation with hand grip strength. The association between grip strength and protein carbonylation in centenarians’ plasma samples was tested using a Pearson correlation in the whole cohort (*p* = 0.07; r = −0.17; n = 108).

Oxidative stress markers were estimated in plasma samples from centenarians via the indirect method of protein carbonylation. As anticipated, we noted a negative trend (n = 108; *p* = 0.07) between protein carbonylation and grip strength when analyzing the entire cohort (Supplementary Table S4 and Figure 7). This trend was also observed in males (n = 24; *p* = 0.10; r = −0.33), but not when the female cohort was studied separately. Furthermore, no significant differences were observed when investigating the association between ADL disability score and protein carbonylation.

### 3.6. BDNF Association with Physical Abilities in Centenarians

**Figure 8:**
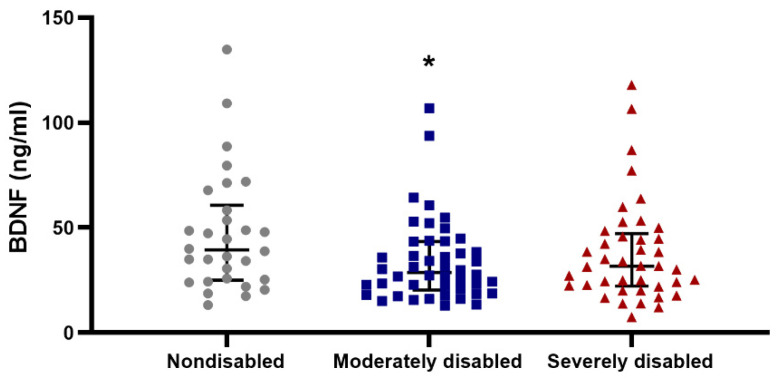
BDNF level is negatively associated with ADL disability score. The association between both ADL disability score and BDNF in women (n = 84) and men (n = 24) was tested using a Kruskal–Wallis test (*p* = 0.06) followed by Dunn’s multiple comparison test with Bonferroni correction (* indicates differences to nondisabled).

BDNF has garnered significant attention due to its apparent correlation with physical exercise [29]. Upon analysis of BDNF levels in the plasma of centenarians, we observed a negative trend (*p* = 0.06; n = 108) between ADL disability score and BDNF levels in these individuals (Figure 8). Notably, centenarians classified as nondisabled showed higher BDNF levels compared to those classified as moderately and severely disabled. However, this trend was not evident when comparing BDNF levels with grip strength (Supplementary Table S4).

## 4. Discussion

In this study of a cohort of Danish centenarians, we find that mitochondrial respiration parameters ATP-linked OCR, Maximal Respiration, Reserve Capacity and Non-mitochondrial OCR in the individual’s PBMCs were higher in the most disabled centenarians. A significant positive correlation was also observed for grip strength of female centenarians regarding mtDNA copy number in their PBMCs. In line with this, mtDNA copy number associated negatively with ADL disability score. Furthermore, activity of the core BER enzyme, APE1 in PBMCs, tended to positively associate with grip strength in females. Lastly, we also observed a trend of negative association between the score of disability and levels of plasma BDNF. The ADL disability score tended to associate positively with the level of plasma carbonylation. Finally, our studied cohort showed a significant positive correlation between physical and cognitive measurements, in agreement with previous studies performed in old individuals [2,6,45].

It is well established that mitochondrial bioenergetics decline with aging. However, literature regarding mitochondrial functioning in very long-lived individuals such as centenarians is sparse. We have previously shown that bioenergetics is well preserved in PBMCs of centenarians [7] and this has also been observed for fibroblasts [46,47]. Age-associated decline in mitochondrial function has been associated with a decline in physical function [48]. However, until now it has not been known whether there is a correlation between physical function and mitochondrial functioning in very old individuals, as they represent a selective group which may exhibit extraordinary aging characteristics.

Here we observed that PBMCs from moderately and severely disabled centenarians (according to their ADL disability score) show higher values of mitochondrial respiration for all mitochondrial parameters, except basal respiration and proton leak. Conversely, we did not find any correlations when mitochondrial respiration was compared to grip strength. To our knowledge, this is the first study investigating the relationship between mitochondrial respiration and physical maintenance with functional disability in centenarians. Association of blood biomarkers, such as serum albumin, serum lipid as well as lymphocyte count, and functional disability (measured by ADL score) has been reported in centenarians previously [49], but mitochondrial function was not considered in that study. Physical disability measured as ADL disability score as well as loss of grip strength have been proposed as important risk factors that affect the future cognitive decline in older persons [50,51]. In line with this, we observed an inverse association between ADL disability score and MMSE score in our cohort, although some variation has also been noted. Despite the MMSE test being commonly used for testing cognition in old age populations [52,53], the associations between cognitive scores and other parameters are complex to address due to the influence of other factors, such as educational level or lifestyle factors, among others. In the case of very old age populations, a passive lifestyle could even exacerbate the cognitive deterioration.

Accordingly, in a previous study performed on the same centenarian cohort [7], the association between mitochondrial respiration and cognitive scores, both CCS and MMSE, also showed that mitochondrial respiration was higher in male centenarians with lower cognitive values. An increase in mitochondrial activity has also been related to neurodegenerative disorders [54,55] and the experimental moderation of mitochondrial respiration has been reported to have beneficial effects when applied in mice [56]. This highlights the role of mitochondrial dysfunction as an important player in both physical and cognitive decline. Functional disability and mitochondrial function in PBMCs have been studied mainly in old individuals. For instance, some studies have shown that fatigue is associated with lower mitochondrial respiration in PBMCs [57]. Others have reported that higher mitochondrial respiration associates with higher gait speed, grip and knee extension strength as well as lower inflammatory status [17]. It is important to keep in mind that the cohort we have studied here consists of a selective group of individuals who have reached an extremely high age. Special characteristics in centenarians’ mitochondria have been reported [46] and this could explain the difference to studies performed with younger elderly. 

We also assessed mtDNA copy number in PBMCs and, we found that a lower mtDNA copy number was associated with a higher ADL disability score and a lower grip strength in female centenarians. Our findings agree with other studies performed in old individuals; thus, an association between lower mtDNA copy number and frailty was reported [20] as well as with high mortality and poor health [19] and lower self-rated health status [58]. The fact that the association between mtDNA copy number and grip strength was only observed in females in our study may be due to the low number of males. It could also be due to sex-dependent differences in the regulation of mitochondrial physiology, as suggested in previous studies [59,60].

Interestingly, we have not observed a correspondence between mtDNA copy number and respiratory activity of mitochondria when comparing to physical activity. The dynamic nature of mitochondrial biology should be considered and changes in mitophagy (clearance of damaged mitochondria), mitochondrial biogenesis as well as a more efficient organization of supercomplexes [46] might explain the differences observed regarding mitochondrial parameters. 

APE1 plays a central role in the DNA Base Excision Repair (BER) pathway of oxidative DNA lesions. It also works as a co-transcriptional activator by modulating gene expression and it controls intracellular redox by inhibiting ROS production [61]. In a previous study performed on this cohort of centenarians, we observed a positive correlation between APE1 expression and activity in their PBMCs and cognitive scores, measured as MMSE [7]. In the current study, we observed a tendency of a positive association between APE1 activity and grip strength in female centenarians, but this was not observed when compared to functional disability. Most studies have focused on the role of APE1 on cognitive function and neurodegenerative diseases and there is scarce literature on the role of this enzyme (and the BER pathway) on physical disability despite the important role of maintenance of genomic stability in aging. One of these few studies is in line with our findings as the authors report an association between increased DNA damage formation (including oxidative stress and abasic sites), defective repair of double-strand breaks and frailty [23]. Our results indicate that APE1 may play an important role not only in maintaining cognitive function but also in maintaining physical function. As APE1 is a versatile enzyme with multiple functions, it is yet unclear by which function APE1 is associated with physical function. However, we consider that, as we have observed only differences regarding APE1 activity but not APE1 expression it may be related to removal of oxidative damage or redox functions. In agreement with that, we have observed a tendency towards a negative association between protein carbonylation in plasma, an indicator of oxidative damage to proteins, and grip strength, which indicates the important role of oxidative damage and its removal for optimal physical functioning. An inverse association between protein carbonylation and grip strength as well as other frailty indicators has also been observed in other studies performed in old individuals [62] and it is consistent with the presumed mechanism by which the accumulation of oxidative damage may contribute to physical decline. 

Brain-derived neurotrophic factor (BDNF), an exercise-induced neurotrophin, has been extensively studied due to its important role in memory consolidation and cognitive function [63]. Recent studies have shown that BDNF levels in plasma can be a predictor for cognitive impairment at very old age and in early stages of AD and dementia [7,64]. In the current study, we observed that levels of BDNF in plasma tended to associate negatively with functional disability in centenarians. In line with our findings, an association between low plasma BDNF levels and frailty in community dwelling older adults has been reported [30]. Circulating BDNF levels may reflect BDNF secreted by the brain in response to exercise as well as other potential sources including platelets, vascular endothelial and smooth muscle cells and activated macrophages and lymphocytes [65,66,67]. Accordingly, it has also been described that blood BDNF levels are associated with muscle strength and physical performance in patients with heart failure [29] and in patients with haemodialysis [68]. Conversely, we have not observed any association between BDNF levels and grip strength. However, the studies mentioned before were based on a small number of subjects and very specific groups with pathologies. Interestingly, BDNF has been suggested as an important regulator of the expression of genes involved in BER, especially APE1 [26,69]. Accordingly, we found similar patterns of association when we compared BDNF and ADL disability score as well as when we compared APE1 activity and grip strength. 

Lastly, NAD+/NADH levels were investigated in our cohort of centenarians and compared to functional disability and grip strength. In brief, we did not see any significant associations. Numerous studies have given much attention to NAD+/NADH levels because of their prominent role in aging and age-related disease [70] as well as their connection to cognitive function [7,71,72]. Loss of muscle mass during aging has also been associated with lower NAD+ levels in skeletal muscle [73] and NAD+ boosting strategies have emerged due to their potential to improve muscle function and mitigate age-associated physiological decline in mice [74]. Our lack of association between NAD+/NADH levels and physical maintenance may mirror aspects of this selective cohort. It would also be interesting to assess NAD levels in other related tissues, and it is important to consider exploring NAD+ instead of NAD+/NADH levels and related metabolites in future studies.

Our study has certain limitations. First, the number of male participants is relatively low, and this may explain why we did not observe significant correlations for some of the parameters in the male cohort. Secondly, it is a cross-sectional analysis, which implies that causality cannot be established. In addition, some of the parameters were measured in PBMCs that comprised a mixture of lymphocytes and monocytes, which may be relevant for some aspects. At the same time, our study’s strengths include a well-characterized population of centenarians and a consistency in the results when compared to previous studies performed in this cohort [7].

## 5. Conclusions

To conclude, our results show that physical disability in centenarians is associated with increased mitochondrial respiration, decreased mtDNA copy number and lower APE 1 activity, as well as lower BDNF plasma levels and high protein carbonylation. Therefore, our findings suggest that key hallmarks of aging also seem to be relevant among centenarians. Future studies may examine the potential role of other mechanisms involved in mitochondrial physiology, such as mitophagy and mitochondrial biogenesis as biomarkers for physical decline.

## Figures and Tables

**Table 1:** Characteristics of the study population: Danish centenarians from the 1915 cohort.

Centenarians	
Population number, n	185
Blood samples, n (%)	135 (73%)
Age, mean (SD)	100.1 (0.04)
Sex frequency, n (%)	
Women	105 (77.8%)
Men	30 (22.2%)
ADL disability score	
Completion frequency, n (%) *	135 (100%)
Score range	1 (Nondisabled), 2 (Moderately disabled), 3 (Severely disabled)
Nondisabled	
n (%)	37 (27.4%)
Moderately disabled	
n (%)	51 (37.8%)
Severely disabled	
n (%)	47 (34.8%)
Grip strength	
Completion frequency, n (%) *	126 (93.3%)
Score range	2–30
Mean (SD)	12.38 (5.07)
Median (IQR)	12 (6)

Abbreviations: IQR, interquartile range; SD: standard deviation. * Completion frequency was different depending on the test performed and it is relative to the number of blood samples.

## Data Availability

The original contributions presented in the study are included in the article/Supplementary Materials. Further inquiries can be directed to the corresponding authors.

## Supplementary Materials

The following supporting information can be downloaded at: https://www.mdpi.com/article/10.3390/biom14080909/s1, Table S1: Association between physical parameters and sex. Table S2: Association between grip strength and mitochondrial function. Table S3: Association between mitochondrial function, ADL disability score and sex. Table S4: Association between molecular markers and grip strength within the 1915 cohort.
