# Supplementary material for: Markers of Mitochondrial Function and DNA Repair Associated with Physical Function in Centenarians
Source: Biomolecules. 2024 Jul 26;14(8):909. doi: 10.3390/biom14080909 (PMC11353237; doi:10.3390/biom14080909)
Supplement: Supplementary file 1 [file biomolecules-14-00909-s001.zip › biomolecules-3081047-supplementary.pdf]

Supplementary Materials

Supplementary Table S1. Association between physical parameters and sex

	**Sex**					**ADL disability score**	
	P-value	Females		Males		Coefficient p-value	Adjustment by sex
**Grip strength**	**<0.001**	11.03 (3.75)		16.89 (6.26)		-2.46 **<0.001**	-2.13 **<0.001**
**ADL disability score**	0.20	n.d.	25 (67.6%)	n.d.	12 (32.4%)		
		m.d.	41 (80.4%)	m.d.	10 (19.6%)		
		s.d.	39 (83%)	s.d.	8 (17%)		

Comparison of grip strength and sex has been tested using a t-test. Means ± S.D. are shown. Comparison among the physical parameters and after adjustment by sex has been tested using a linear regression. Coefficient and p value are shown.
Comparison of ADL disability score and sex has been tested using a χ^2 test. n.d.=nondisabled; m.d.=moderately disabled; s.d.= severely disabled.

Supplementary Table S2. Association between grip strength and mitochondrial function

	Grip strength					
	**Both sexes**		**Female**		**Male**	
	**r**	**p-value**	**r**	**p-value**	**r**	**p-value**
**Basal respiration**						
Pearson correlation	0.0006	0.99	0.03	0.78	-0.13	0.52
Spearman rank correlation	-0.02	0.78	-0.01	0.90	-0.11	0.57
**Proton Leak**						
Pearson correlation	0.02	0.80	0.09	0.42	-0.14	0.48
Spearman rank correlation	0.005	0.95	0.08	0.43	-0.15	0.46
**ATP linked OCR**						
Pearson correlation	-0.003	0.97	0.02	0.84	-0.13	0.53
Spearman rank correlation	-0.04	0.66	-0.04	0.74	-0.09	0.64
**Maximum respiration**						
Pearson correlation	0.04	0.67	-0.04	0.70	0.07	0.72
Spearman rank correlation	-0.003	0.97	-0.07	0.49	0.10	0.62
**Reserve capacity**						
Pearson correlation	0.06	0.48	-0.02	0.80	0.09	0.64

Spearman rank correlation	0.02	0.80	-0.04	0.71	0.14	0.50
**Non mitochondrial OCR**						
Pearson correlation	-0.06	0.48	-0.07	0.51	-0.09	0.63
Spearman rank correlation	-0.12	0.21	-0.14	0.18	-0.11	0.58
**Basal ECAR**						
Pearson correlation	-0.14	0.14	-0.09	0.39	-0.25	0.22
Spearman rank correlation	-0.14	0.13	-0.10	0.35	-0.35	0.08
**Maximal ECAR**						
Pearson correlation	-0.05	0.55	0.02	0.81	-0.33	0.10
Spearman rank correlation	-0.09	0.33	-0.03	0.76	-0.35	0.08

Basal ECAR was also measured as part of our protocol and a measure of glycolytic reserve capacity (also called Maximal ECAR) was obtained after addition of oligomycin. Units for Basal respiration, Proton leak, ATP linked OCR, Maximal respiration, Reserve capacity and non mitochondrial respiration are: pmol/min/µg protein. Units for Basal ECAR and Glycolytic reserve capacity are: mpH/min/ µg protein

Supplementary Table S3. Association between mitochondrial function, ADL disability score and sex

	**Association between mitochondrial function and ADL disability score**		**Adjustment by sex**	
	Coefficient	p-value	Coefficient	P-value
**Basal Respiration**	0.79	0.14	0.83	0.10
**Proton Leak**	-0.03	0.63	-0.03	0.65
**ATP linked OCR**	**1**	**0.03**	**1.00**	**0.03**
**Maximal Respiration**	**4.46**	**0.02**	**4.58**	**0.03**
**Reserve capacity**	**3.6**	**0.03**	**4.18**	**0.01**
**Non mitochondrial OCR**	**0.37**	**0.01**	**0.38**	**0.01**
**Basal ECAR**	**0.2**	**0.04**	**0.19**	**0.05**
**Maximal ECAR**	0.03	0.86	0.06	0.76
**Mitochondrial DNA copy number**	**-6.55**	**0.05**	**-8.33**	**0.004**

A quantile regression of the median has been performed between mitochondrial function and ADL disability score and after an adjustment for sex. Units for Basal Respiration, Proton Leak, ATP linked OCR, Maximal Respiration, Reserve capacity and non mitochondrial respiration are pmol/min/µg protein. Units for Basal ECAR and Glycolytic reserve capacity are: mpH/min/µg protein.

Supplementary Table S4. Association between molecular markers and grip strength within the 1915 cohort.

	**Grip strength**					
	**Both sexes**		**Female**		**Male**	
	**r**	**p-value**	**r**	**p-value**	**r**	**p-value**
**APE 1 activity**						
Pearson correlation	0.16	0.12	**0.21**	**0.07**	0.02	0.92
Spearman rank correlation	0.15	0.15	**0.20**	**0.08**	0.09	0.71
**APE1 protein**						
Pearson correlation	-0.01	0.90	-0.08	0.41	0.10	0.63
Spearman rank correlation	-0.12	0.21	-0.16	0.13	-0.06	0.76
**NAD+/NADH**						
Pearson correlation	-0.13	0.19	-0.08	0.44	-0.001	0.99
Spearman rank correlation	-0.11	0.23	-0.10	0.36	-0.04	0.82
**BDNF**						
Pearson correlation	-0.07	0.42	-0.04	0.71	-0.16	0.43
Spearman rank correlation	-0.03	0.75	-0.02	0.8	-0.09	0.64
**Protein Carbonylation**						

Pearson correlation	**-0.17**	**0.07**	-0.12	0.24	-0.33	**0.10**
Spearman rank correlation	-0.12	0.18	-0.09	0.39	-0.31	0.13

Units for APE1 protein level and protein carbonylation are A.U. (arbitrary units), for APE1 activity are amol/ng/min, for NAD+/NADH are µM and for BDNF are ng/mL

Supplementary information: APE1 protein determination in whole cell extract from PBMCs

WCE samples (2 µg) were incubated at 95°C for 5 min in 1XNuPage LDS Loading Dye (Thermo-scientific) containing 100 mM DTT and separated on 4–12% NuPAGE Novex® Bis-Tris gels (Invitrogen, Denmark) in 1XMES running buffer (Invitrogen, Denmark) for 20 min at 80V and 2 hrs at 120V. Proteins were transferred to a polyvinylidene fluoride (PVDF) membrane by dry transfer using the iBlot 2 dry blotting system (Thermo Scientific) (transfer program: 20V for 1 min, 23V for 4 min, 25V for 2 min). The membrane was blocked in TBS-T (20 mM Tris (pH 8.0), 137 mM NaCl, 0.05% Tween) with 5% w/v non-fat milk followed by incubation with appropriate primary antibodies: anti-APE1 (1:1000, Thermo #PA5-29157) and mouse anti-β-actin (1:20,000, Sigma #A2228), respectively. Subsequently, the membrane was washed in TBS-T and thereafter with appropriate specific secondary antibody for 1 h at room temperature. Secondary antibodies were: horseradish peroxidase-linked anti-rabbit IgG (1:10,000, GE Healthcare #NA934) and anti-mouse IgG (1:10,000, GE Healthcare #NA931), respectively. Proteins were visualized by the enhanced chemiluminescence procedure (ECL plus®; GE Healthcare, Amersham, The Netherlands). For control of loading, the proteins of interest were normalized to the signal for β-actin. One or two internal control(s) were loaded on all gels and all measurements were normalized to the internal controls to reduce inter-assay and loading variation. Band intensities were quantified by ImageJ software.
